# Exploration of Intrinsic Microbial Community Modulators in the Rice Endosphere Indicates a Key Role of Distinct Bacterial Taxa Across Different Cultivars

**DOI:** 10.3389/fmicb.2021.629852

**Published:** 2021-02-16

**Authors:** Pei Wang, Xiao Kong, Hongsong Chen, Youlun Xiao, Huijun Liu, Xiaojuan Li, Zhuo Zhang, Xinqiu Tan, Diandong Wang, Decai Jin, Ye Deng, Tomislav Cernava

**Affiliations:** ^1^College of Plant Protection, Hunan Agricultural University, Changsha, China; ^2^Hunan Plant Protection Institute, Hunan Academy of Agricultural Sciences, Changsha, China; ^3^School of Public Health, Qingdao University, Qingdao, China; ^4^Key Laboratory of Environmental Biotechnology, Research Center for Eco-Environmental Sciences, Chinese Academy of Sciences, Beijing, China; ^5^Guangxi Key Laboratory for Biology of Crop Diseases and Insect Pests, Institute of Plant Protection, Guangxi Academy of Agricultural Sciences, Nanning, China; ^6^Beijing Key Laboratory of Detection and Control of Spoilage Organisms and Pesticide Residues in Agricultural Products, Beijing University of Agriculture, Beijing, China; ^7^College of Life Science and Technology, Yangtze Normal University, Chongqing, China; ^8^College of Resources and Environment, University of Chinese Academy of Sciences, Beijing, China; ^9^Institute of Environmental Biotechnology, Graz University of Technology, Graz, Austria

**Keywords:** *Oryza sativa*, phyllosphere, endosphere, microbial community, plant-microbe interactions

## Abstract

Microbial communities associated with the plant phyllosphere and endosphere can have both beneficial as well as detrimental effects on their hosts. There is an ongoing debate to which extend the phyllosphere and endosphere microbiome assembly is controlled by the host plant how pronounced cultivar effects are. We investigated the bacterial and fungal communities from the phyllosphere and endosphere of 10 different rice cultivars grown under identical environmental conditions in the frame of a targeted approach to identify drivers of community assembly. The results indicated that the endophytic bacterial communities were clearly separated into two groups. The α-diversity and microbial network complexity within Group I were significantly lower than in Group II. Moreover, the genera *Nocardioides*, *Microvirga*, and *Gaiella* were significantly more abundant in Group II and only present in the interaction networks of this group. These three genera were significantly correlated with α- and β-diversity of the endophytic bacterial community and thus identified as major drivers of the endosphere community. We have identified keystone taxa that shape endophytic bacterial communities of different rice cultivars. Our overall findings provide new insights into plant-microbe interactions, and may contribute to targeted improvements of rice varieties in the future.

## Introduction

With only a few rare exceptions, plants are generally associated with highly diversified microbial communities (Heil, 2015; Manzotti et al., 2020). These microorganisms are closely associated with ecosystem functions, which can be either beneficial or harmful (Graham et al., 2016). The phyllosphere of plants is likely the largest microbial habitat on earth (Redford et al., 2010) with a global leaf surface area estimated to be ∼6.4 × 10^8^ km^2^ and contains up to 10^26^ bacteria (Lindow and Brandl, 2003; Vorholt, 2012). This habitat constitutes an oligotrophic environment and microorganisms that colonize it are affected by many factors, such as temperature, UV radiation, growing season, CO_2_ concentration, which all affect microbial communities to a certain extend (Remus-Emsermann et al., 2012; Copeland et al., 2015; Ren et al., 2015; Pan et al., 2019). In addition to microbes that colonize plant surfaces, many microorganisms, collectively referred to as endophytes, are also found within plant tissues.

Plant endophytes are associated with almost every plant on earth and fulfill various essential role therein (Strobel et al., 2004). These specific microorganism usually colonize root hairs, leaves, vascular tissues, epidermal cells, intercellular spaces, and the cytoplasm (Strobel et al., 2004). Endophytes use roots, flowers and natural openings on the leaves and stem lenticels as entry points to inner plant tissues (Lodewyckx et al., 2002; Kandel et al., 2017). In general, endophytes that grow within healthy plants often contribute with certain functions to their host’s growth and health for a mutual benefit. They can increase host disease resistance through the secretion of bioactive compounds (Busby et al., 2016; Matsumoto et al., 2021), augment nitrogen transfer from soil to plant (Behie and Bidochka, 2014), as well as improve crop performance (Liu et al., 2017).

Plant endophytes provide the basis for a broad range of applications in agriculture. They can be used to increase plant biomass, improve plant health, or degrade environmental contaminants (James et al., 2002; Sandhu et al., 2007; Puri et al., 2016; Feng et al., 2017a). In contrast, pathogenic microorganisms that can occur in the same niches in plants can cause various plant as well as human diseases (De Kempeneer et al., 2004; Teplitski et al., 2011). Therefore, a better understanding of indigenous plant-associated microbial communities will likely provide the means to counteract harmful aspects that are accompanied by pathogen invasion.

Rice (*Oryza sativa* L.) is the most commonly cultivated crop in China and also serves as a staple food in many other countries of the world. There are thousands of different rice varieties in the world, with new cultivars continuously arising from extensive breeding approaches. While phenotypic traits can substantially differ between cultivars, there is contradicting evidence to which extend the plant-associated microbiome is determined by the plant genotype. For example, Hunter et al. demonstrated that lettuce genotypes significantly affect phyllosphere bacterial and fungal communities (Hunter et al., 2010; Hunter et al., 2015). However, Lundberg et al. (2012) showed that the host genotypes had a weak effect on the root-associated microbiota of *Arabidopsis thaliana*. In terms of rice plants, there is currently no consensus on whether the plant genotype affects the associated microbial communities and to which extend they are affected. Hardoim et al. (2011) demonstrated that the formation of bacterial communities associated with rice roots was affected by the genotype. Different plant genotypes can have diverging compositions of their root exudates, which influences microorganisms in the rhizosphere. Interestingly, Wu et al. (2009) demonstrated that variations in the rice cultivar and nitrogen fertilizers only influenced methanogens that are tightly associated with rice roots. Due to the high variations in the plant phyllosphere, there is an ongoing debate on whether the microbiome assembly in this microenvironment is controlled by the host genotype. The intrinsic potential of microbial communities in the phyllosphere to modulate the community structure and composition was so far mostly ignored.

In this study, 10 rice cultivars were grown within the same plot and under the same management conditions to explore the relationship between plant cultivar and leaf-associated microbial communities. The bacterial and fungal communities from the phyllosphere and endosphere of these cultivars were analyzed by high-throughput sequencing of marker genes. The aims of the study were as follows: (1) To decipher the differences in the cultivars’ phyllosphere communities; (2) to investigate the potential of each cultivar to shape the phyllosphere microbiome; (3) to reveal potential keystone species that influence the rice leaf-associated microbiome.

## Materials and Methods

### Site Description and Sample Collection

All samples for this study were collected from an experimental field of the Rice Blast Identification Center in Taojiang, Hunan, China (112°06′34″E, 28°38′55″W). The 10 rice cultivars that were implemented in this study are described in Table 1. All cultivars were planted in one experimental field, separated by ditches, and received the same management program (including irrigation, fertilization, and pesticide treatements). The soil is a fertile, heavy loam in which rice can be grown during the whole year. The samples were collected at the tillering stage with no visible occurrence of pests or plant diseases.

**TABLE 1:** Rice cultivars implemented in the present study.

**One-letter code**	**Cultivar**	**Hybrid information (female parent × male parent)**
A	Quanyou 248	Quan 9311A × R248
B	Quanyou 636	Quan 9311A × R636
C	Tyou 272	T98A × Huahui 272
D	Liangyou 0293	P88S × 0293
E	Quanyou 631	Quan 9311A × R631
F	Fengliangyou 4	Feng 39S × Yandao 4
G	Fengliangyou 406	Feng 39S × R406
H	Shenliangyou 857	Shen 08S × R857
J	Shenliangyou 523	Shen 08S × R523
K	Shenliangyou 837	Shen 08S × R837

*The one-letter codes are used throughout the manuscript for the designated cultivars.*

Five leaves from each plant and six replicates for each cultivar were randomly collected and placed in an isolated box with cooling packs for transport to the laboratory. The total community DNA from phyllosphere epiphytes and endophytes was collected as follows. The leaves were immersed in sterile PBS buffer (0.02 mM, pH 7.0, 0.1% Tween 80), and shaken on a thermostatic oscillator (BSD-YF2600, Shanghai Boxun Medical Biological Instrument Crop., Shanghai, China) for 30 min at 30°C, followed by 10 min of sonication (Ningbo Xingzhi Biological Technology Co., Ltd., Ningbo, China). To enrich epiphytic phyllosphere microorganisms the suspension was passed through a 0.22 μm membrane using a vacuum pump, the membrane was then stored at 4°C until DNA extraction.

Once phyllosphere microorganisms were collected the leaves were surface sterilized with 75% ethyl alcohol for 3 min, 2.5% sodium hypochlorite (NaClO) for 5 min, and washed with sterile water five times to remove residual reagents. The leaves were then ground with a sterile mortar and pestle in PBS, silica sand was used to increase the friction. The resulting supernatant was stored at 4°C until DNA extraction.

### Total Community DNA Extraction and PCR Amplification of Marker Genes

Genomic DNA was extracted with the Fast DNA spin kit for soil (MP Biomedicals LLC, United States) according to the manufacturer’s instructions. The 16S rRNA gene was amplified with primer pair 799F (AACMGGATTAGATACCCKG)/1115R (AGGGTTGCGCTCGTTG) (Kong et al., 2018), while the ITS region of fungi was amplified with primer pair gITS7F (GTGARTCATCGARTCTTTG)/ITS4 (TCCTCCGCTTATTGATATGC) (Wei et al., 2018). Each primer pair contained a unique barcode (12 bp) to distinguish different samples after sequencing. The PCR reaction was as follows: 5 μL 10 × PCR buffer, 4 μL dNTP, 0.5 μL DNA polymerase (TaKaRa Biotech, Beijing, China), 1.5 μL each primer (10 μM) and 1 μL DNA template. The PCR thermocycling conditions were as follows: Initial denaturation at 94°C for 1 min, denaturation at 94°C for 20 s, annealing at 57°C for 25 s, and extension at 72°C for 45 s, the denaturation, annealing and extension processes were repeated 30 and 35 times for the amplification of 16S rDNA and ITS target fragment, respectively. A final extension at 72°C for 10 min was conducted for both reactions. The PCR products were visualized by electrophoresis in a 2% agarose gel and purified with E.Z.N.A. Gel Extraction Kit (Omega Bio-tek, Norcross, GA, United States) following the manufacturer’s instructions. Sequencing was conducted by the commercial provider Annoroad Gene Technology (Beijing, China), and the Illumina Miseq platform for 2 × 250 bp paired-end sequencing. A total of 240 samples was sequenced in this study (phyllosphere and endosphere samples, bacterial and fungal samples, ten cultivars, six replicates; 2 × 2 × 10 × 6 = 240).

### Analysis of High-Throughput Sequence Data and Statistical Analysis

The raw data was subjected to initial processing with the online pipeline^1^ (Feng et al., 2017b). In the first step, the forward and reverse sequence files were merged into full length sequences by using FLASH (Magoc and Salzberg, 2011). Reads with average quality score <20 or containing ambiguous nucleotides (N) were removed from the analysis. Then, sequences were trimmed based on their length. All bacterial 16S rRNA gene fragment sequences with a length <295 or >305 bp were removed. For the fungal ITS region reads with <300 or >400 bp were removed. An OTU (operational taxonomic unit) table was generated at 97% similarity level with the UPARSE algorithm (Edgar, 2013). For the fungal analysis, the ITS sequences were verified with the ITSx tool in order to remove other eukaryotic sequences (Bengtsson-Palme et al., 2013). Taxonomic assignments were conducted with the RDP database and the Warcup database (Wang et al., 2007). Finally, to account for variations in sequencing depth, the OTU tables were resampled randomly and the resulting tables were used for downstream analysis. The sequencing data is publicly available at the NCBI Sequence Read Archive under accession no. SRP230471 (PRJNA586741).

Before deepening analyses were conducted, it was confirmed that the rarefaction curves were saturated which suggested that the sequencing depth was sufficient to reflect the microbial community (Supplementary Figure 1). The alpha diversity of microbial communities was assessed with the Shannon index. The community composition was analyzed at different phylogenetic levels (phylum and genus) and the beta diversity was visualized with a principal coordinate analysis (PCoA) based on a weighted UniFrac matrix. Dissimilarity tests based on the multi-response permutation procedure (MRPP), adonis (PERMANOVA), and analysis of similarities (ANOSIM) were used to evaluate differences among groups. Functional groups were identified with the FAPROTAX (Functional Annotation of Prokaryotic Taxa) (Louca et al., 2016) and FunGuild bioinformatic tools (Nguyen et al., 2016) for the bacterial and fungal communities, respectively. A Mantel test was used to calculate the correlation between environmental factors and the observed microbial community.

### Construction of Microbial Interaction Networks

Interaction networks were constructed to assess the interaction between different taxa in the rice phyllosphere. For fungi, only an epiphyte network was constructed due to the limited number of replicates for fungal endophytes after the removal of samples with insufficient read numbers. The networks were constructed with an online pipeline available at http://ieg4.rccc.ou.edu/mena (Deng et al., 2012). First, the resampled OTU tables of each rice cultivar were used to construct individual networks. The filter was set to 80%, so that only operational taxonomic units (OTUs) present in a minimum of five of the six replicates were considered. Logarithmic data transformation was omitted and correlation calculations were performed with Spearman’s rho. The networks were constructed based on the RMP method with a threshold (cutoff) of 0.91, 0.95, and 0.86 for phyllosphere bacteria, endophytic bacteria, and phyllosphere fungi, respectively. Network properties, such as average connectivity, average path length, and average clustering coefficient (avgCC) were separately calculated for each dataset. Finally, the networks were visualized with Cytoscape 3.3.0 (Shannon et al., 2003).

### Statistical Analyses

The statistical analyses were carried out with IBM SPSS statistics 21. Significance tests were performed with one-way ANOVA and the correlation analysis was carried out by assessing Pearson’s correlation coefficient.

## Results

### General Data Statistics and Microbial Community Diversity

Following initial quality filtering, several samples within the fungal endophyte dataset contained <10,000 reads after quality control, therefore only three replicates were available for the downstream analysis of most cultivars. In total 7,166,642 bacterial and 7,780,134 fungal high-quality sequences were retained after quality control. Moreover, ranges of 76 – 4,540 and 39 – 386 OTUs per sample were obtained after taxonomic classification for bacteria and fungi, respectively. Following resampling for data normalization of 16S rDNA gene fragment and ITS region samples, 10,635 and 11,711 reads per sample were retained, respectively.

**FIGURE 1:**
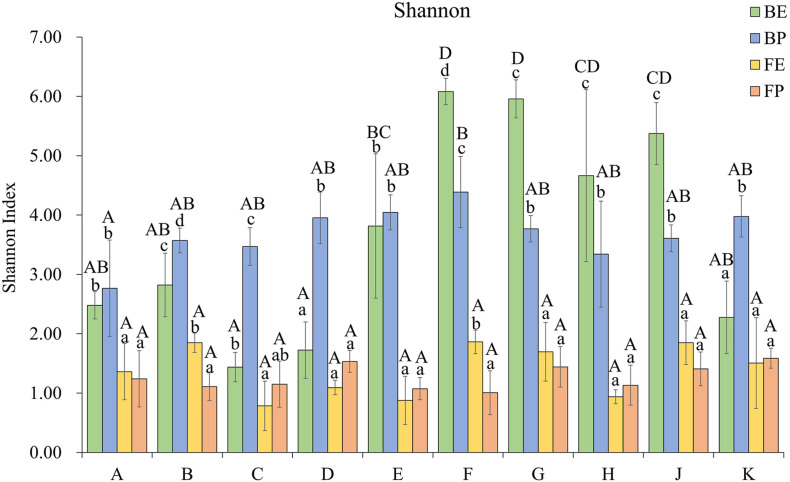
Shannon indices of phyllosphere and endosphere microbial communities from ten different rice cultivars. BE, endophytic bacteria; BP, phyllosphere bacteria; FE, endophytic fungi; FP, phyllosphere fungi. The capital letters designate significant differences (*P* < 0.05) between different cultivars and the lowercase letters represent significant differences (*P* < 0.05) among different samples of the same cultivar (BE, BP, FE, and FP).

The Shannon index indicated differences in microbial diversity of different samples (Figure 1). Among the endophytic bacterial community samples, cultivars E, F, G, H, and J (Group II) harbored more diverse communities than Group I comprising cultivars A, B, C, D, and K (*P* < 0.05). Among all samples, cultivar F was associated with the highest diversity (H’ = 6.08) while cultivar C (H’ = 1.44) had the lowest alpha diversity. For phyllosphere and endophytic fungal communities, no significant difference was found among the cultivars. Within the Group II cultivars, the order of diversity was: endophytic bacteria ≥ phyllosphere bacteria > endophytic fungi ≥ phyllosphere fungi, while for Group I cultivars, the diversity was: phyllosphere bacteria ≥ endophytic bacteria > endophytic fungi ≥ phyllosphere fungi.

### Microbial Community Composition of Rice

The bacterial community was primarily composed of the phyla Proteobacteria (53.01–97.21%), Actinobacteria (0.16–27.56%), and Firmicutes (0.68–13.19%) while the fungal community was dominated by Basidiomycota (77.11–100%) and Zygomycota (0–18.33%) (Supplementary Figure 2). The relative abundance of Actinobacteria and Firmicutes in the endosphere of Group II was higher than in Group I. Zygomycota were only present in endosphere of cultivars A, B, G, H, J, and K. The fungal community of phylloshere samples was almost entirely composed of the phylum Basidiomycota (99.88–100%).

**FIGURE 2:**
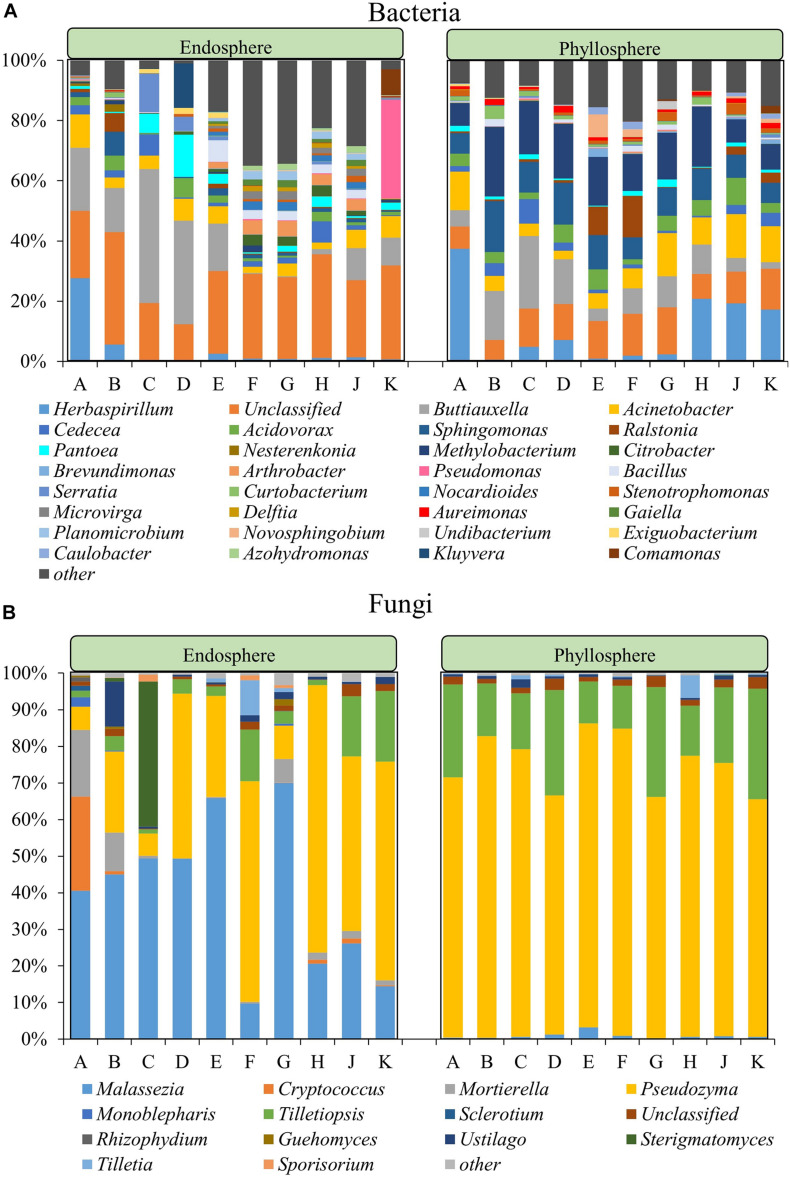
Community composition within the bacterial **(A)** and fungal **(B)** community at genus level. For both communities the phyllosphere and the endosphere are separately shown. The fraction labeled with “other” represents the sum of all microorganisms with a relative abundance <2% in all community types.

In a deepening assessment the microbial community composition was assessed at genus level for all datasets. The endophytic bacterial community compositions of Group II cultivars were more similar to each other while the same trend was observed within Group I (Figure 2A). Within Group I, there were six bacterial genera in common for all cultivars, including *Acinetobacter*, *Buttiauxella*, and *Serratia*, while a total of 93 genera were in common for Group II, including the highly abundant *Bacillus*, *Sphingomonas*, *Pantoea*, and *Nocardioides*. Six genera, *Acinetobacter*, *Buttiauxella*, *Cedecea*, *Pantoea*, *Serratia*, and *Sphingomonas*, were present in all 10 rice cultivars. Significance analyses for differential abundance indicated that members of the genera *Buttiauxella* were significantly more abundant in the endosphere of Group I while *Citrobacter*, *Arthrobacter*, *Nocardioides*, *Microvirga*, *Delftia*, *Gaiella*, *Planomiicrobium*, *Undibacterium*, and *Azohydromonas* were significantly more abundant in the endosphere of Group II (*P* < 0.05). Overall, the species richness of Group II was higher than Group I, while the abundance was lower. In the bacterial phyllosphere community, a total of 14 genera, including *Acinetobacter*, *Buttiauxella*, *Acidovorax*, *Sphingomonas*, *Cedecea*, and *Methylobacterium*, were detected in all 10 rice cultivars. In addition, members of the genus *Herbaspirillum* were more abundant in cultivars A, H, J, and K. *Buttiauxella* was more abundant in cultivars B–D while *Ralstonia* was more abundant in cultivars E and F. The relative abundance of other bacteria showed no significant difference among the different cultivars.

The fungal genera *Malassezia*, *Moesziomyces*, and *Tilletiaria* were present in the endosphere and phyllosphere (epiphytes) of all ten rice cultivars. The endophytic fungal community was dominated by *Pseudozyma* (6.08–73.11%), *Malassezia* (9.76–70.03%) and *Mortierella* (0.11–18.20%) while the epiphytic phyllosphere fungal community was dominated by *Pseudozyma* (64.99–3.98%) and *Tilletiopsis* (11.46–30.12%) (Figure 2B). The relative abundance of *Malassezia* in the endosphere of cultivars F and K was lower than in the other cultivars. The relative abundance of *Sterigmatomyces* in the endosphere of cultivar C and *Rhizophydium* in the endosphere of cultivar A was higher than in the other cultivars. Within the phyllosphere samples, the relative abundance of *Malassezia* was significantly higher in cultivar E and *Tilletiopsis* was higher in cultivars D, G, and K when compared to other cultivars.

### Microbial Community Structures Among Different Cultivars and Sample Types

**FIGURE 3:**
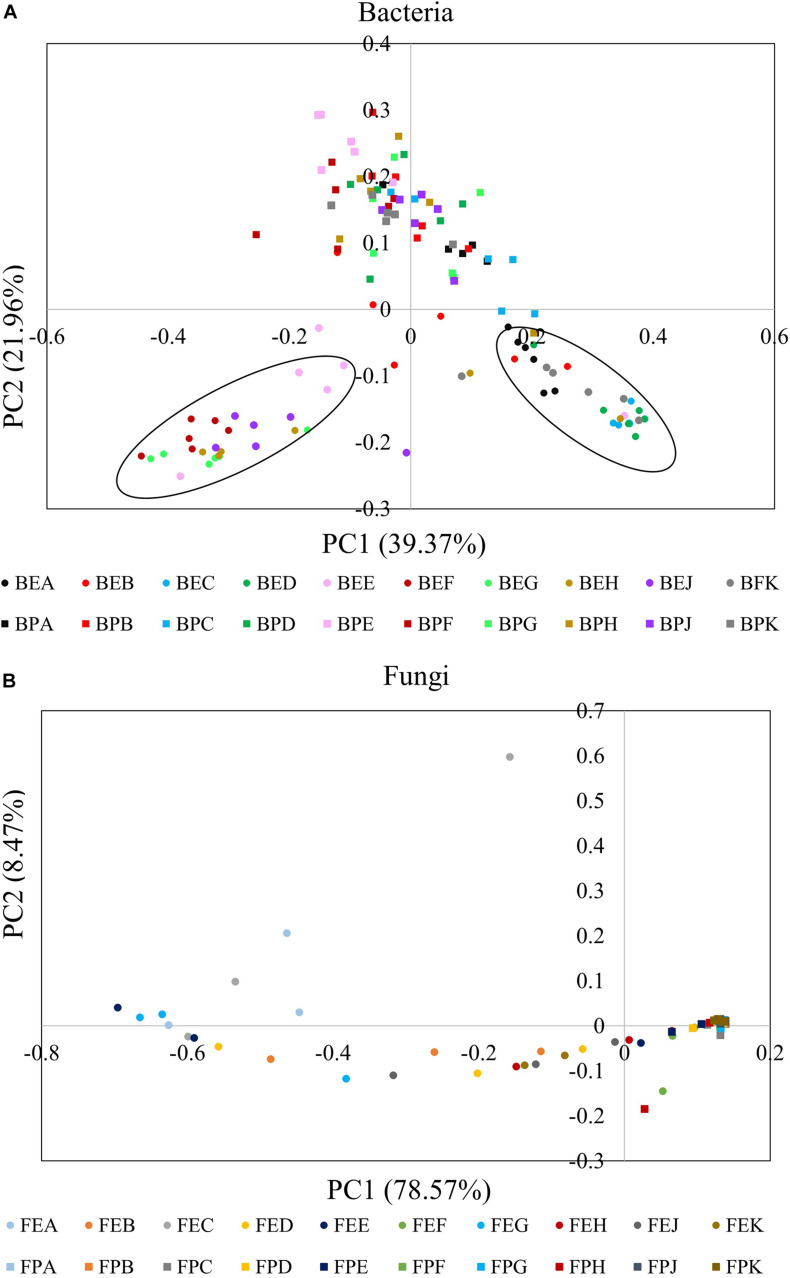
Principal coordinate analysis (PCoA) plots based on weighted UniFrac matrix of bacterial **(A)** and fungal communities **(B)**. The initial letters B and F stand for bacterial and fungal, respectively. The middle letters E and P stand for endosphere and phyllosphere, respectively. The last letter A–K designate the implemented cultivars.

A PCoA plot based on a weighted UniFrac matrix was used to visualize differences in community structures (Figure 3). The first axis (PC1) accounted for 39.37 and 78.57% difference while the second axis (PC2) accounted for 21.96 and 8.47% of the difference for bacterial and fungal communities, respectively. The phyllosphere and endophytic communities, both bacterial and fungal, were significantly different from each other. Also, the endophytic bacterial communities of the ten cultivars clearly clustered into two groups; Group I: cultivars A, B, C, D, and K and Group II: cultivars E, F, G, H, and J. The community structure of the phyllosphere bacteria, phyllosphere fungi, and endophytic fungi showed no significant difference among the cultivars.

### Identification of Community Modulators via Microbial Interactions Networks

Different networks were constructed in order to infer intra-community interactions. Following initial assessments, only networks of the endophytic bacterial communities were further analyzed, because no significant differences were found in the networks from phyllosphere samples. The overall network topologies are shown in Table 2. The number of total nodes, total links, average degree (avgK), and number of modules in Group II were significantly higher than in Group I, indicating that cultivars E, F, G, H, and J hosted more complex networks. The degree of distribution in these networks fitted well with the power law (*R*^2^ of power law: 0.309 – 1), suggesting that these networks have scale-free properties, which indicates that most nodes have few neighbors while a few nodes have many neighbors. The avgCC and modularity in these empirical networks were significantly different from their corresponding random networks, which confirmed that these networks were not random. The average path lengths (GD) were within the range of 1.091 to 9.237, which were close to the logarithmic values of the total number of network nodes, an indication that these nodes were closely related to each other, and that the networks showed small-world properties. Modularity ranges from 0.504 to 0.857, indicated that these networks could be divided into modules and that a module was a functional unit.

**TABLE 2:** Topological properties of the empirical molecular ecological networks (MENs) and their associated random MENs.

**Cultivar/Network Index**	**Empirical networks**								**Random networks**		
	**Similarity threshold**	**Total nodes**	**Total links**	***R*^2^ of power-law**	**Average degree (avgK)**	**Average clustering coefficient (avgCC)**	**Average path distance (GD)**	**Modularity (the number of modules)**	**Average clustering coefficient (avgCC)**	**Average path distance (GD)**	**Modularity**
A	0.95	55	77	0.634	2.8	0.413	1.883	0.58 (16)	0.074 ± 0.025	3.278 ± 0.169	0.499 ± 0.019
B	0.95	29	33	0.762	2.276	0.51	1.672	0.708 (8)	0.061 ± 0.033	3.720 ± 0.383	0.543 ± 0.028
C	0.95	16	10	1	1.25	0.188	1.091	0.82 (7)	0.188 ± 0.000	1.522 ± 0.180	0.779 ± 0.029
D	0.95	10	6	1	1.2	0.076	1.444	0.666 (4)	0.096 ± 0.014	1.902 ± 0.325	0.764 ± 0.050
E	0.95	174	793	0.309	9.115	0.677	3.583	0.588 (23)	0.135 ± 0.014	2.739 ± 0.038	0.247 ± 0.007
F	0.95	742	1648	0.829	4.442	0.407	9.237	0.814 (81)	0.009 ± 0.002	4.339 ± 0.027	0.476 ± 0.005
G	0.95	689	1934	0.771	5.614	0.433	8.46	0.751 (66)	0.017 ± 0.003	3.826 ± 0.024	0.398 ± 0.004
H	0.95	225	1644	0.378	14.613	0.683	4.292	0.504 (14)	0.179 ± 0.010	2.477 ± 0.022	0.184 ± 0.005
J	0.95	585	1352	0.763	4.622	0.445	8.743	0.815 (66)	0.013 ± 0.003	4.150 ± 0.030	0.460 ± 0.005
K	0.95	37	33	0.875	1.784	0.378	1.267	0.857 (13)	0.035 ± 0.018	4.053 ± 0.602	0.680 ± 0.032

Complementary *Z-P* plot analyses were implemented to further define the roles of individual nodes within the networks (Supplementary Figure 3). The network nodes were grouped according to four possible roles: peripherals, connectors, module hubs, and network hubs. In this study, the majority of nodes were assigned to peripherals (99.06%), while connectors and module hubs, were only found in cultivars E, F, G, and J, where they made up 0.39 and 0.55% of the nodes, respectively. No network hubs were found at all. The connectors and module hubs were assigned to Proteobacteria (*Sphingomonas* sp., *Filomicrobium* sp., *Microvirga* sp., and *Shinella* sp.), Actinobacteria (*Blastococcus* sp., *Arthrobacter* sp., *Gaiella* sp., and *Bryobacter* sp.), and Firmicutes (*Cohnella* sp.).

**FIGURE 4:**
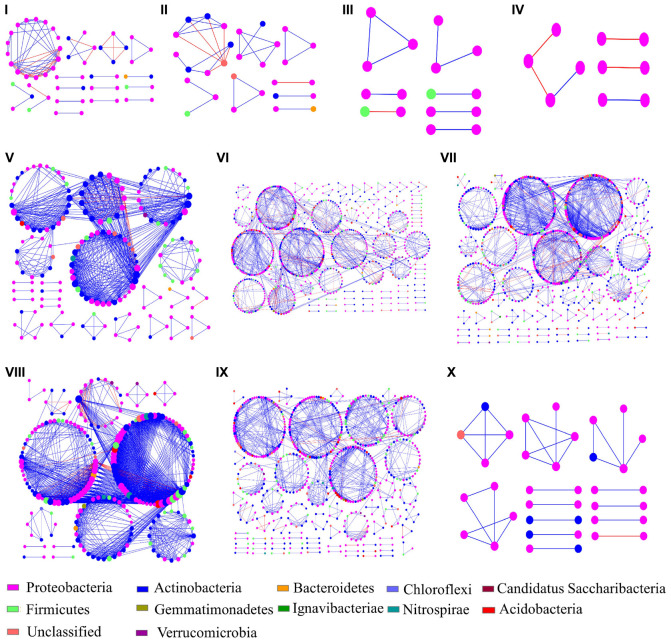
Bacterial endophyte interaction networks of ten rice cultivars. The different sections **(A–J)** represent the cultivars **(A–H,J,K)**, respectively. The node colors represent different phyla and the node size represent the number of links with other nodes. Blue and red links indicate positive and negative interactions, respectively.

The ten networks mainly consisted of Proteobacteria (46.3 – 100%), which was also the only identified phylum present in cultivar D (Figure 4). Actinobacteria were the second most abundant phylum in the obtained networks (16.22 – 30.46%), except for cultivars C and D. The relative abundances of Proteobacteria and Actinobacteria were higher and lower, respectively, in Group II as compared to Group I. The ratio of positive interactions in Group II was also higher than in Group I. At the genus level, *Nocardioides* was the most abundant in the networks of Group II while it was not found in the networks of Group I. In addition, the genera *Bacillus*, *Gaiella*, *Microvirga*, *Arthrobacter*, *Massilia*, and *Blastococcus* were also predominant in the networks of Group II. On the contrary, the composition of Group I networks showed substantially less complexity with *Herbaspirillum*, *Sphingomonas*, *Cedecea*, *Acinetobacter*, and *Methylobacterium* as the dominant genera in different networks.

### Identification of Keystone Taxa That Affect the Endophytic Bacterial Communities

Genera with relative abundance higher that 1% were implemented in correlation analyses with α-diversity of endophytic bacterial communities among different cultivars. The results indicated total 24 genera were significantly associated with α-diversity. These bacteria were further used to analyze the correlation with endophyte community structures. This resulted in ten genera, whereof three genera were also shown to be part of the microbial networks indicating intra-community interactions. These three genera were assigned to *Nocardioides*, *Gaiella*, and *Microvirga*, and their relative abundances in Group II (0.18–4.38, 0.85–2.98, and 0.86–2.82%) were substantially higher than that in Group I (0–0.03%, 0.01–0.13%, and 0–0.08%, respectively). The three genera showed a significantly positive correlation with α- and β-diversity (*P* < 0.05, Table 3). In a complementary assessment that included the plant phenotype, the leaf length was found to be negatively correlated with the relative abundance of these three genera (*P* < 0.05).

**TABLE 3:** Identified keystone bacterial genera that affect the endophytic bacterial community across different rice cultivars.

	***Nocardioides***	***Microvirga***	***Gaiella***
Leaf length	−0.441**	−0.480**	−0.495**
α-diversity	0.655**	0.703**	0.709**
β-diversity	0.093*	0.105*	0.131*

**indicates significant difference at 0.01 level, *indicates significant difference at 0.05 level.

## Discussion

Microbial communities within the plant phyllosphere and endosphere play an important role in promoting the growth and health of their hosts (Ren et al., 2015). In this study, analyses of microbial community compositions and network interactions within the phyllosphere and endosphere of 10 different rice cultivars were conducted.

While the phyllosphere epiphytic communities showed no clear trends, the endophytic bacterial communities of the analyzed rice cultivars were clearly separable into two groups. The diversity of the endophytic bacterial community in Group I (cultivars A, B, C, D, and K) was significantly lower than that of Group II (cultivars E, F, G, H, and J). This finding is similar to an observation by Jiang et al. (2017) in which the rhizosphere bacterial communities of blueberry cultivars clearly clustered into three groups. In general, higher diversity in a biological system correlates with higher resistance to environmental changes (Loreau and de Mazancourt, 2013), which suggests that the microbial ecosystem stability of Group I is lower than that of Group II. In addition, we observed that the diversity of the phyllsophere bacterial community in Group I was higher than the endophytic bacterial community, while in Group II the opposite trend was found. The observed differences between bacterial communities in the phyllosphere and the endosphere might be due to differing permeability of the cultivars for colonization by endophytes. However, no significant difference was found in the diversity of fungal communities located in the phyllosphere and endosphere among the ten cultivars.

The relative abundance of the bacterial phyla Actinobacteria and Firmicutes in the endosphere was significantly higher in Group II then compared to Group I. Although Group I contained fewer species, the genera *Herbaspirillum*, *Buttiauxella*, and *Acinetobacter* were more abundant therein. For Group II, a higher number of species was detected, but no dominant genus for the entire group was found. *Herbaspirillum*, is a Gram-negative bacterial genus that is commonly found in the endosphere of rice. On one hand, it was found to be a plant-growth promoting bacterium, while on the other hand it can also act as a mild pathogen that impairs rice growth by augmenting ethylene levels (Valdameri et al., 2017). In addition, nitrogen fixation is characteristic for this genus (Chubatsu et al., 2011). The genus *Buttiauxella* belongs to the Enterobacteriaceae family and was previously found in the endosphere of rice seeds (Wang et al., 2020). It was shown to improve plant growth and to enhance the remediation efficiency for cadmium (Cd) in the host plant (Wu et al., 2018). Various *Acinetobacter* species are known to exert plant growth-promoting effects and decrease adverse effects of PAHs in rice (Gao et al., 2006).

When fungal communities were analyzed, the endosphere of the ten cultivars was dominated by *Malassezia* and *Pseudozyma* while the phyllosphere fungal community was dominated by *Pseudozyma* and *Tilletiopsis*. *Pseudozyma* sp. is commonly found on leaves of rice and other plants (Kitamoto et al., 2011; Nasanit et al., 2015). It can provide protection against plant-pathogenic fungi by producing distinct extracellular fatty acids (Avis and Bélanger, 2002). *Tilletiopsis* spp. were previously found in intercellular fluid from leaf blades and sheaths of rice, and they were shown to be promising biocontrol agents for the control of powdery mildew (Haggag et al., 2015). In the present study, the relative abundance of potentially beneficial microorganisms differed among rice cultivars, indicating that their ecological implications might be redundant, i.e., replaceable by other community members.

In the last decade, various studies reported that plant cultivars shape their associated microbiomes. For example, Manter et al. (2010) demonstrated that the endophyte bacterial community in potato roots is cultivar-dependent. Analogous to this finding, the study of Lopez-Velasco et al. (2011) indicated that the phyllosphere bacterial community of spinach is also determined by the cultivar. Furthermore, Materatski et al. (2019) demonstrated that the plant cultivar is one of the main factors that shapes the endophytic fungal community. However, there is still some controversy regarding the relative importance of the environment in terms of shaping the microbiome when compared to cultivar effects (Morella et al., 2020). For example, Kong et al. (2020) indicated that bacterial and fungal communities located in the phyllosphere of three different maize cultivars showed no significant difference. Singh et al. (2019) demonstrated that the phyllosphere microbial community of grapes was affected by the grape species and the growing year, with the latter having a more pronounced effect. In the present study, in order to exclude the impact of environmental factors, all 10 rice cultivars were grown under identical environmental conditions in the same field. Our results indicated that the differences among phyllosphere bacterial, phyllosphere fungal, and endophytic fungal communities were not statistically significant. However, the endophytic bacterial community was separable into two groups, with cultivars A, B, C, D, and K (Group I) and cultivars E, F, G, H, and J (Group II) clustering closer together and showing the same trends in their α-diversity. The genotypic relatedness of the different cultivars could not explain the group formation. For example, cultivar H and cultivar K, cultivar A and cultivar E, were derived from the same female parent, but their endophytic bacterial communities were significantly different. This is in contrast to cultivars of other plants where endophytic communities were shown to correspond to the genotype (Abdullaeva et al., 2020; Chen et al., 2020). Our observation therefore indicated that the analyzed rice cultivars were not the main drivers of the structure of leaf-associated microbial communities. In order to explore intrinsic factors within microbial communities, interaction networks of endophytic bacterial communities were screened for keystone taxa.

Networks analyses are suitable to infer interactions among different microbial species, such as competition and mutualism (Zhou et al., 2011; Deng et al., 2012). In the present study, the interaction networks of bacterial endosphere communities showed that the network complexity of cultivar Group I was significantly lower than Group II, indicating that the microbial inter-connectivity within Group II was higher than within Group I. In addition, *Nocardioides*, *Microvirga*, and *Gaiella* were found as the most important members within interaction networks of cultivar Group II while they were not present in networks of Group I. *Nocardioides* sp. is a common endophyte and has been isolated from the endosphere of many plants, such as *Jatropha curcas* L. (Qin et al., 2012), maize (Glaeser et al., 2014; Kampfer et al., 2016), mugwort and horse-weed (Han et al., 2013), *Carex scabrifolia* Steud (Song et al., 2011), and *Perilla frutescens* (Du et al., 2013). *Nocardioides* sp. contains dozens of species and some of them exhibit plant growth-promoting activities through nitrogen fixation (Nafis et al., 2019). Other species, such as *N. alkalitolerans*, *N. jensenii*, *N. rotundus*, and *N. mesophilus* are capable of reducing nitrate (Toth et al., 2008; Dastager et al., 2010; Wang et al., 2016). Representatives of *Microvirga* were also isolated from some plant tissues, such as the root endosphere of rapeseed (Jimenez-Gomez et al., 2019), roots endosphere of *Abulitonindicum* (Bhadania et al., 2019), as well as the rice rhizosphere (Li et al., 2020). Moreover, *Microvirga* spp. were also found to be nitrogen-fixing and plant growth-promoting bacteria (Jimenez-Gomez et al., 2019; Zhao et al., 2019). *Gaiella* spp. were previously detected in the endosphere of *Lolium perenne* L. and found to play an important role in the plant’s interaction network (Wemheuer et al., 2017). Hu et al. (2020) detected *Gaiella* spp. in bulk soil and rhizosphere soil of tobacco. In addition, *Gaiella* can reduce nitrate to nitrite (Albuquerque et al., 2011) which is essential for nitrogen cycling. These three genera were significantly correlated with both α- and β-diversity of the endophytic bacterial communities indicating a key role in community modulation. While direct cultivar effects on microbial communities can be low in distinct plants, we hypothesize that a low number of host-selected microbes (e.g., *Nocardioides*, *Microvirga*, and *Gaiella* found in in Group II of the present study) can exert substantial intra-community modulating effects. Host-microbe interplay was further confirmed in the present study by correlation analyses between the relative abundance of the three identified community-shaping genera and their host plant’s leaf length. The results indicated distinct phenotypic adaptions of the host in response to these taxa. Further research will be required to decipher the underlying mechanisms and to identify potential effects on the overall host performance as a response to these microbes.

## Conclusion

Our study provides important clues related to the assembly of microbial communities in rice. It was found that 10 rice cultivars can be either assigned to a group with microbial communities of relatively low complexity (Group I) or to a group with relatively high complexity (Group II). Correlation analyses revealed that the genera *Nocardioides*, *Microvirga*, and *Gaiella* significantly correlated with the α-diversity as well as the β-diversity of the endophytic bacterial communities. The relative abundance of the three genera was significantly higher in Group II where they were found to be embedded in complex intra-community interaction networks, which is indicative for an important role in community modulation. In summary, our results indicate that the plant cultivar can enrich distinct endophytes in certain cases, which then exert further modulating effects on the remaining microbial community. To the best of our knowledge, this is the first study to comprehensively investigate plant microbial communities among different rice cultivars and mechanistically uncover potential causes for community variance.

## Data Availability Statement

The datasets presented in this study can be found in online repositories. The names of the repository/repositories and accession number(s) can be found below: https://www.ncbi.nlm.nih.gov/genbank/, SRP230471.

## Author Contributions

All authors listed have made a substantial, direct and intellectual contribution to the work, and approved it for publication.

## Conflict of Interest

The authors declare that the research was conducted in the absence of any commercial or financial relationships that could be construed as a potential conflict of interest.

## Footnotes

1 http://mem.rcees.ac.cn:8080
