# Supplementary material for: Exploration of Intrinsic Microbial Community Modulators in the Rice Endosphere Indicates a Key Role of Distinct Bacterial Taxa Across Different Cultivars
Source: Front Microbiol. 2021 Feb 16;12:629852. doi: 10.3389/fmicb.2021.629852 (PMC7920960; doi:10.3389/fmicb.2021.629852)
Supplement: Supplementary file 1 [file Data_Sheet_1.DOCX]

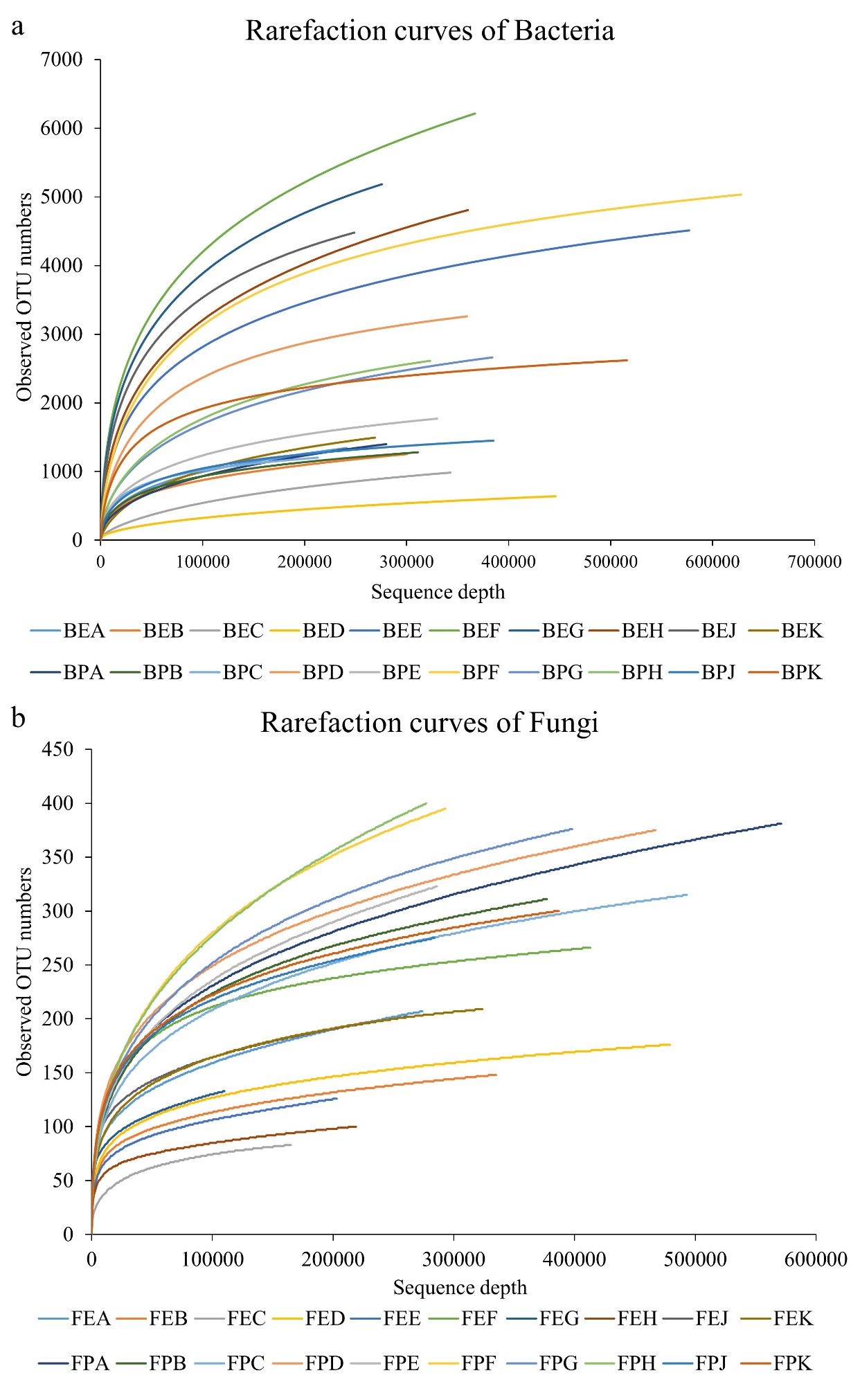


Figure S1 Rarefaction curves of bacterial (a) and fungal communities (b) from different rice cultivars. The initial B and F stand for bacterial and fungal, respectively. The middle letter E and P stand for endosphere and phyllosphere, respectively. The last letter A-K stand for the cultivars.


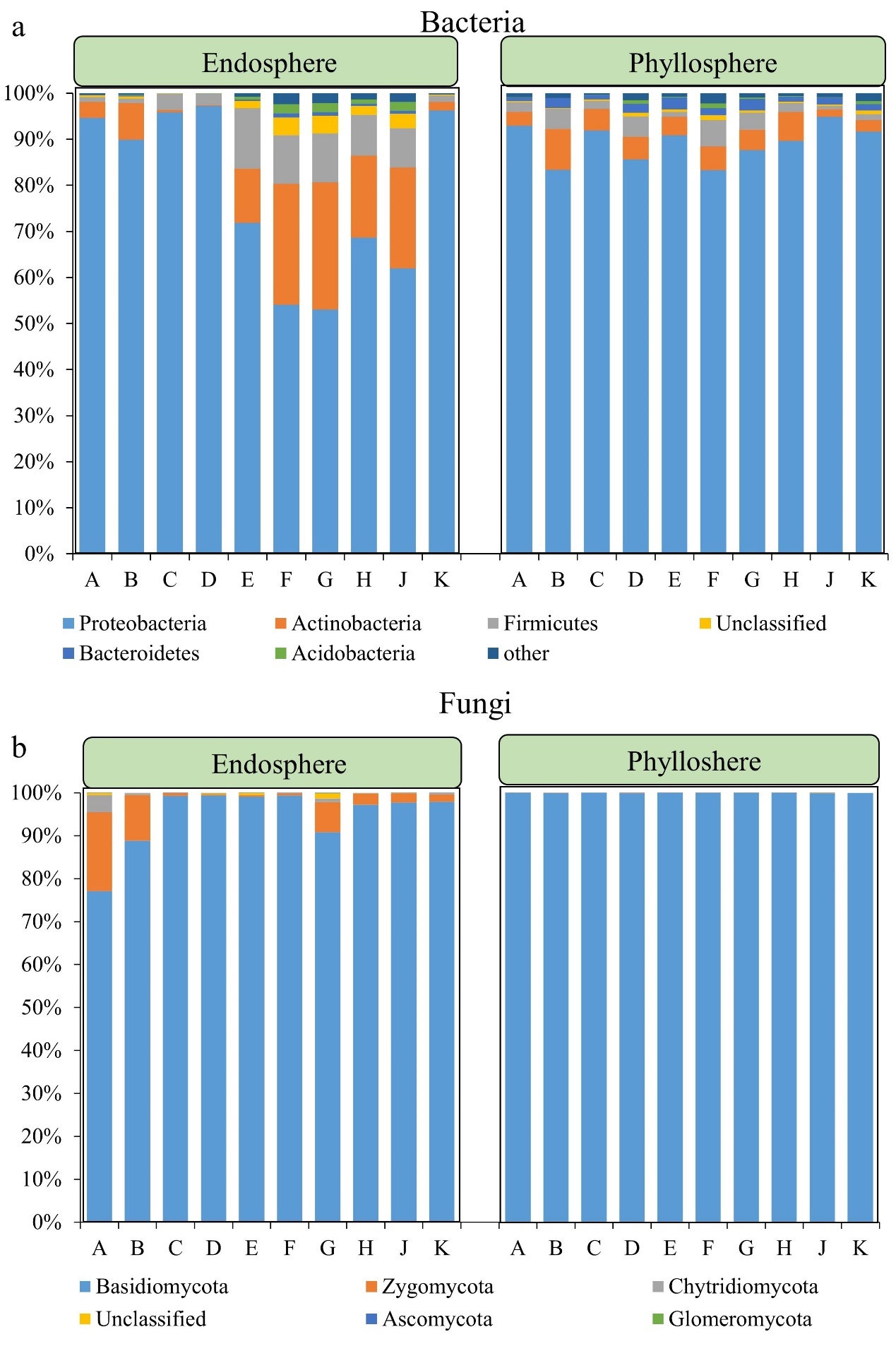


Figure S2 Relative abundance of microbial communities at phylum level.


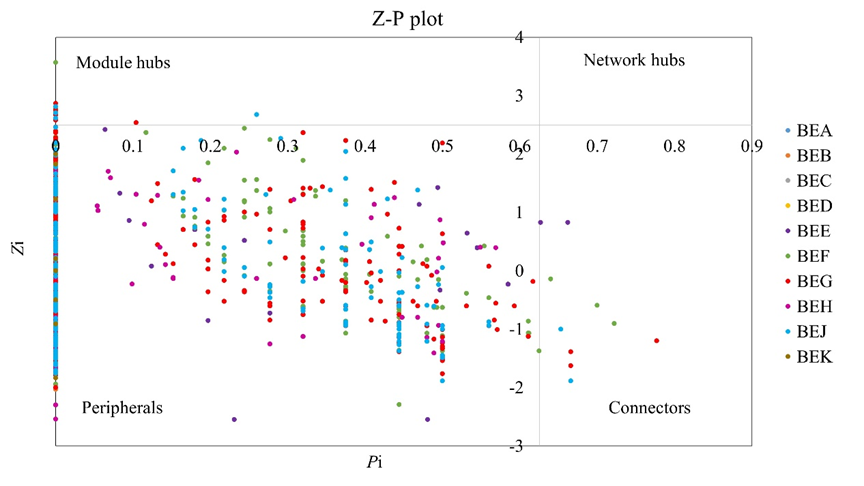


Figure S3 Z-P Plot showing the module-based topological role for each node. The initial B stand for bacterial, the middle letter E stand for endosphere, and the last letter A-K stand for the cultivars.


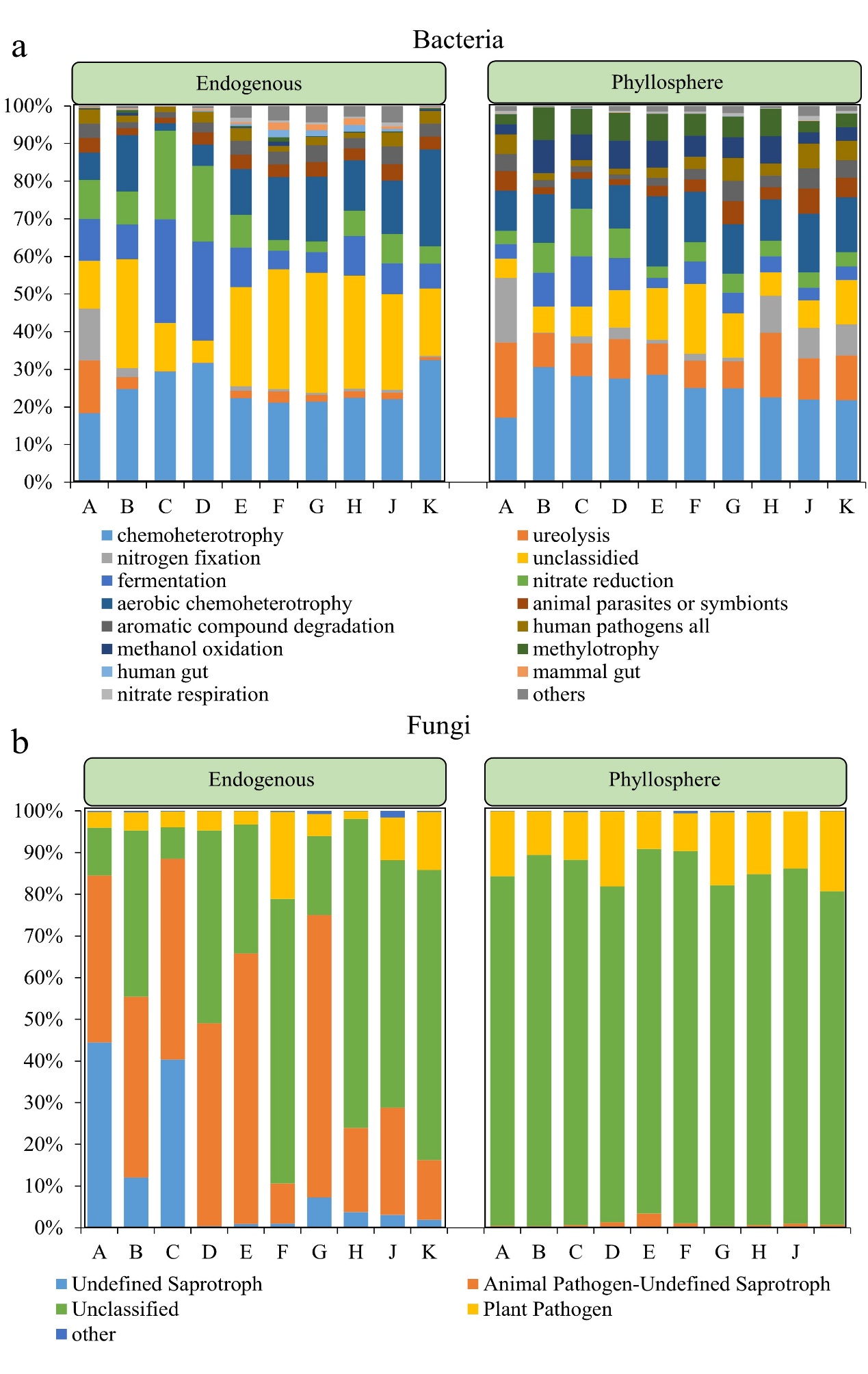


Figure S4 Functional annotation of bacterial (a) and fungal (b) communities based on PFEPROTAX and FunGuild.
